# The impacts of innovation capability and social adaptability on undergraduates’ employability: The role of self-efficacy

**DOI:** 10.3389/fpsyg.2022.954828

**Published:** 2022-11-17

**Authors:** Xiang Li, Ruihui Pu, Hong Liao

**Affiliations:** ^1^School of Foreign Languages and Cultures, Panzhihua University, Panzhihua, China; ^2^Faculty of Economics, Srinakharinwirot University, Bangkok, Thailand

**Keywords:** innovation capability, social adaptability, self-efficacy, employability, undergraduates, higher education

## Abstract

**Introduction:** As the world is consistently driven by the infusion of new-generation information technology and the knowledge economy, college students are placed under mounting pressure in developing occupation-related competencies. Their employability has been receiving growing concerns from stakeholders such as higher education institutions, governments, employers, parents, and even student groups themselves as it plays a decisive role in occupational success, social stability, and economic prosperity. Under the theoretical guidance of social cognitive theory, this study set out to investigate the cognitive and psychological mechanisms through which innovation capability, social adaptability, and self-efficacy influence the employability of college students. It also attempts to analyze the mediating role of self-efficacy in the relations between innovation capability, social adaptability, and employability which has been rarely studied in academia.

**Methods:** A quantitative approach was employed in this study. Data was collected from 726 undergraduates from 9 higher education institutions in the mainland of China by questionnaire survey method. The research model showed a good fit (χ2/df=4.46, RMSEA=0.069, SRMR=0.049, GFI=0.934, CFI=0.965, NFI=0.955, TLI=0.955). Structural equation modeling (SEM) was applied to this study for data analysis.

**Results:** The findings showed that innovation capability, social adaptability, and self-efficacy significantly and positively correlates with undergraduates’ employability. University students with stronger innovation capability, social adaptability, and self-efficacy tend to be more employable in the job market. Model 4 of SPSS PROCESS Macro revealed that self-efficacy played a mediating role in the correlation between innovation capability, social adaptability, and employability.

**Discussion:** Undergraduates with higher levels of innovation capability and social adaptability are more confident in their abilities to take specific actions and achieve expected goals, which in turn intensifies their employability. The study suggests the possibility of improving undergraduates’ employability through positive interference of innovation capability, social adaptability, and self-efficacy in the era of information technology and knowledge-based economy.

## Introduction

The unemployment of college graduates has become a growing issue raising concerns with governments, universities, and society in general (Green and Henseke, 2016; Minocha et al., 2017; Mok et al., 2021). Only through employment can today’s graduates from higher education institutions earn an income, learn work values, and find personal development that will lead to success and prosperity. From a macro perspective, employment also helps maintain social stability (Crowley, 2016; Roy and Das, 2019) and boost economic growth (Seyfried, 2011; Ren et al., 2022). In many countries, the cultivation of students’ occupation-related competencies and attributes to improve employment prospects is regarded as a key strategy for the development and transformation of higher education (Sin et al., 2019; Rees, 2021; Healy et al., 2022). However, employers are facing mounting pressure in offering sufficient jobs to graduates. In particular, since the outbreak of the COVID-19 pandemic in 2019, many enterprises have been placed in a terrible predicament due to economic decline (Flögel and Gärtner, 2020), market disruption (Wang et al., 2021) and decreasing sales (Kim D., 2021), and the labor market has been devastated by the reduced demand of employees from industries and businesses and increased supply of talents from higher education institutions, which in turn has made an increasingly severe problem of the employment of graduating college students and those who have recently graduated during this pandemic. Taking China as an example, on November 19, 2021, the Ministry of Education and the Ministry of Human Resources and Social Security pointed out that the number of Chinese college graduates would surge to 10.76 million in 2022, which increased by 1.67 million over the same period last year. Although the majority of colleges and universities have included the cultivation of students’ employability in their pedagogical programs, practitioners in businesses and industries still indicate that graduates turn out to be not work-ready and lack some fundamental skills required by the real job positions (Pauw et al., 2008; White et al., 2021). Related academic studies have also pointed out that graduates have insufficient skills valued in modern work circumstances (Sarkar et al., 2020; Mgaiwa, 2021). The main crux of the current employment difficulties of college graduates can be attributed to the lack of employability (Cumming, 2010) because such ability enables them to be more employable in the labor market and increase the possibility of finding a job.

Employability refers to the combination of personal characteristics such as ability, personality, desire, and social resources to secure employment, including the knowledge and skills possessed by individuals in the process of developing their careers, as well as a series of comprehensive adaptations to the work contexts (Schreuder and Coetzee, 2006; Yorke, 2006; Zhang et al., 2022). It is the embodiment of comprehensive competencies to drive career development based on learning ability. In the interconnected and ever-changing working environment, employability is a critical factor for both organizations that want to gain competitive advantage and individuals who are pursuing career success (Fugate et al., 2004). Organizations need to ensure that there are enough employable talents who have the ability, motivation, and quality to maintain the survival and development of the organization. Graduates expect to acquire employable qualities to serve the changing contemporary workplaces and meet the requirements of internal transformation in higher education (Wickramasinghe and Perera, 2010). Employability is the comprehensive demand for college students’ career success, enterprise efficiency, and economic development. Bridging the cultivation of applied talents in tertiary education institutions and the demand of the real economy, great attention has been paid to both theoretical and practical viewpoints of major stakeholders including government, employers, colleges, and the students themselves.

Today’s world is experiencing an unprecedented fundamental shift because the mode of economic development has changed from factor-driven and investment-driven to innovation-driven (Xu et al., 2022). Innovation is currently recognized as a key driver of industrial upgrading, economic growth, competitive advantage, and sustainable development (Lewin et al., 2016; Mazzucato, 2018; Chen et al., 2020). Countries all over the world are formulating policies to promote innovation-driven development to sustain and enhance the national core competitiveness and comprehensive strength, such as reindustrialization in the United States, Industry 4.0 in Germany, and Made in China 2025 (Wang and Dong, 2022). Cultivating innovative talents turned into a common goal of higher education institutions because the development of students’ innovative capability can further enhance the strategic advantages and survivability of organizations and countries (Jingyu and Su, 2021). Enterprises that are transforming from a labor-intensive growth model to an innovation-driven growth model are in urgent need of innovative talents (Irfan Sabir and Moazzam Sabir, 2010). Despite the fact that innovation capability plays such a critical role in the cultivation of college students, research has mostly been done from the perspectives: (1) the effect of innovation and entrepreneurship education in enhancing undergraduates’ employability (Li, 2017); (2) the pedagogical innovation in improving the employability skills (Martínez-Cerdá et al., 2020); (3) supporting employability by a skills assessment innovative online tool (Gabor and Matis, 2019). However, few studies have been conducted pertaining to how innovation capability of college students impacts their employability.

Social adaptability refers to the practices of adjusting to social institutions and coordinating behavior to accommodate the social environment (Zhang and Xia, 2021). Previous research on the employability of college undergraduates confirmed that adaptability is an influential factor for students to attain a sustainable competitive edge in knowledge and competence (Chung and Chae, 2016). It is noteworthy that most of the related academic efforts have been made in general adaptability (van Dam, 2013; Helens-Hart, 2019; Collie et al., 2020) and career adaptability (Atitsogbe et al., 2019; Al-Jubari et al., 2021; Matijaš and Seršić, 2021; Stead et al., 2022). For example, a cross-sectional study of 405 final-year nursing college students found that career adaptability significantly predicted employability (Ma et al., 2021). Social adaptability, one of the key influencing factors of competitiveness in the job market for college students (Zhang and Xia, 2021), has not been paid due attention by the academic community.

Both innovation capability and social adaptability are highly sought-after abilities placed on the shoulder of college students by modern society. The advancement of science and technologies in various fields such as information, transportation, energy, materials, engineering, sports etc. requires today’s talents to be innovative to follow the pace and lead the development of these new technologies (Tang et al., 2022; Zhang and Liu, 2022). Society expects colleges and universities to develop qualified graduates who have the adaptability and innovation capabilities to be successfully employed (Borg et al., 2019). On the other hand, the constantly changing society poses numerous challenges for college students to be well-adapted for their personal well-being and career success. An ever-changing environment requires them to be adaptable to identify and take advantage of career opportunities (Fugate et al., 2004; González-Romá et al., 2018). Launching social practices and strengthening the social adaptability of college students can improve their employability and help alleviate the current pressure of difficult employment for college students (Qi, 2014). In view of this, the current study investigates the impact of innovation capability and social adaptability on undergraduates’ employability in the same research context.

Under the theoretical guidance of social cognitive theory that elucidates how humans regulate and tune their own behavior (Owusu-Agyeman and Fourie-Malherbe, 2021), the current research sets out to investigate the cognitive and psychological mechanisms (Zhang X. et al., 2021) through which innovation capability, social adaptability, and self-efficacy may influence the employability of college students. According to social cognitive theory (Bandura, 1977, 1986, 1997), although behavior is jointly determined by the external environment and internal cognition, cognition plays a leading role in determining the final action. Cognitive skills contribute to the cultivation of knowledge and intelligence skills (Anderson and Krathwohl, 2001) and are the key elements highlighted in various employability studies (Coetzee and Beukes, 2010; Rees, 2021; Bennett and Ananthram, 2022). The core component of the cognition factor is self-efficacy developed through the interaction between internal personal factors and environmental events, and its formation is influenced by a variety of factors (Myyry et al., 2022). Self-efficacy was found to have a significantly and positively direct influence on undergraduates’ employability (Chow et al., 2019; Sultana and Malik, 2020; Tentama and Nur, 2021) and play a mediating role in the relationship between the transformational leadership of teachers and the employability of students (Wang et al., 2020), between identification with commitment and perceived employability skills (Chukwuedo et al., 2022) and between teacher knowledge transfer and student employability (Zhao et al., 2021). Nevertheless, the mediation role of self-efficacy on the relations between innovation capability and employability and between social adaptability and employability among college students has hardly been studied.

In summary, most of the previous academic efforts have been made in detecting the components of undergraduates’ employability (McQuaid and Lindsay, 2005; Fugate and Kinicki, 2008; Clarke, 2018; Zhang et al., 2022) and exploring the influencing factors such as career adaptability (Udayar et al., 2018; Atitsogbe et al., 2019), self-efficacy (Berntson et al., 2008; Dacre Pool and Qualter, 2013), soft skills (Finch et al., 2013), emotional intelligence (Potgieter and Coetzee, 2013), career satisfaction (Nauta et al., 2009; Dacre Pool and Qualter, 2013), academic performance (Pinto and Ramalheira, 2017), and work-integrated learning (Jackson, 2015), but little research has been done pertaining to how and to which extent the innovation capability and social adaptability of undergraduates affect their employability. In the meanwhile, no previous research has simultaneously taken innovation capability, social adaptability, self-efficacy, and undergraduates’ employability into consideration. The present work attempts to systematically investigate the correlation between these factors and the employability of college undergraduates. Additionally, the research explored the mediating function of self-efficacy on the correlation between innovation capability, social adaptability, and employability. By exploring the correlations and interactions between these constructs, the current study can offer practical implications for governments, tertiary education institutions, and college students as to how to bring about positive interference in improving undergraduates’ employability.

## Literature review and hypothesis development

### Employability

Employability is a multi-dimensional and multi-level construct (Bennett and Ananthram, 2022; Hu et al., 2022; Zhang et al., 2022). However, some definitions have been widely accepted, retested, and updated by researchers in the past two decades. Employability is defined as an integration of capabilities, knowledge, and personal traits that make it easier for individuals to obtain jobs and attain achievements in their selected careers, thus benefiting themselves, the labor market, the economy, and society (Yorke, 2006). It refers to individuals’ abilities such as knowledge, logical thinking, learning quality, self-administration, and interpersonal skills (Honig et al., 2017) to address the needs of employers to the greatest extent and complete the assignments delivered by the employers (Boh et al., 2016). It means continuously completing or obtaining work by making the best utilization of both one’s employment-related abilities and meta-competencies (Heijde and Van Der Heijden, 2006; Schreuder and Coetzee, 2006). By constantly applying and developing a series of supporting capabilities and attributes through dynamic and evolving stages, it increases the opportunities for individuals to obtain and maintain job opportunities (Jackson and Oliver, 2018; Spencer, 2021). Related research on employability has been conducted from different perspectives (individual, organizational, and industrial) by scholars across various academic disciplines such as educational science (Pinto and Ramalheira, 2017; Atitsogbe et al., 2019), business and management studies (Hogan et al., 2013), sociology (Liu et al., 2020), psychology (Vanhercke et al., 2014; Sultana and Malik, 2020) etc. in terms of definition (Fugate et al., 2004; Tomlinson, 2012), model (Knight and Yorke, 2002; Pool, 2017), factors (Nauta et al., 2009; Finch et al., 2013; Udayar et al., 2018) and evaluation (Palmer et al., 2018; Cotronei-Baird, 2020).

For the present study, college students’ employability is a comprehensive professional ability obtained through acquiring knowledge and developing qualities during their studies in colleges and universities, which can realize their values and meet the needs of society by finding jobs after graduation. The employability of college students is not a static concept but a dynamic organism that integrates competencies, knowledge, and psychological attributes acquired in higher education to help them adapt to the ever-changing internal and external work circumstances of the future (Vermeulen et al., 2018). It helps students become work-prepared according to occupational demands and assist employers in providing job applicants with the best chance of sustainable employment (Singh et al., 2017). Involving both employment and career development, it is regarded as a key factor affecting students’ future employment prospects (Thijssen et al., 2008).

### Innovation capability

Innovation refers to the generation and implementation of novel ideas beneficial to the corresponding context (Baumol, 2010) and the execution of notably ameliorated ideas, products, processes, methods, practices, or relations (OECD, 2005). It is a comprehensive ability to fulfill innovative processes and generate innovative results by putting knowledge and skills into use (Hao, 2021). For the most part, innovation and creativity are used as synonyms in academic literature. Innovation is regarded as the realization of creativity by fundamentally reconstructing and re-imagining existing objects and integrating novel ideas and thoughts (Heap, 1989). Innovative ability has turned into one of the most critical employability attributes of college students that help them better prepare for future workplaces (Acar and Tuncdogan, 2019). Innovative college students are characterized by curiosity, associative thinking, bravery, and creative self-efficacy (Hulme et al., 2014). Students’ innovation capability refers to a set of self-perceived abilities and expertise that students can learn and utilize through college curriculum and training courses to better generate innovation results (Shane, 2007; Mars and Hoskinson, 2013). It is regarded as a key higher education outcome that is prioritized as an institutional strategy by educators (Selznick and Mayhew, 2018, 2019). They are driven to formulate strategies to encourage students and teachers to think creatively and identify opportunities through teaching, learning, and research (Binks, 2014). They are also striving to enhance students’ employability by improving their skills, expertise, attitude as well as innovation capability, so as to address the increasingly fierce international challenges (Nanjundeswaraswamy and Swamy, 2022). Innovation education is conducive to cultivating college students’ innovative spirit and ability, which is of great significance to improve their employability (Li, 2017). Students see the ability to innovate as critical to their employability and preparing them for future jobs (Edziwa and Blignaut, 2022). Innovative college graduates find jobs faster than their classmates (Pilav-Velic et al., 2020). Developing students’ innovative spirit and practical competence is an important approach to increasing the employment of graduates and an effective measure to relieve the employment pressure. Developing college students into innovative professionals helps them adapt to the dynamic landscape of the workplace and secures sustainable employment (Chang, 2014). Based on these arguments, Hypothesis 1 was proposed:

*H1*: Innovation capability of college students has a direct and positive effect on their employability.

### Social adaptability

As the world is constantly changing, college students should not merely have solid theoretical knowledge and professional skills, but also be equipped with strong social adaptability. Adaptability refers to all the strategies adopted by a person in order to cope with conflicts in natural and social environments (Sadock et al., 2007), as well as the process of adapting to social systems and adjust their actions to suit the social context (Zhang and Xia, 2021). It is what individuals must acquire in their life to integrate into the external environment and culture and achieve their own physical and mental growth. The process of social adaptability is substantially the process of consecutive socialization of human beings (Helens-Hart, 2019). In addition, it reflects one’s capacity to handle daily affairs and independently assume social responsibilities, as well as whether he/she has acquired the ability to meet sociocultural expectations (Wang et al., 2012). Social adaptability is one of the essential qualifications that college students need in order to participate in social life and the labor market. Developing strong social adaptability can facilitate their smooth entry into society, sound interpersonal relationships, and career success in the end. Based on an extensive meta-analysis of 202 studies, adaptability steadily demonstrated the strongest association with perceived employability among students (Harari et al., 2021). Only with strong social adaptability can college students overcome all kinds of hindrances at work and keep moving forward in the difficulties they may encounter in the future (Kovess-Masfety et al., 2016). Graduates in social education who have stronger adaptability to new situations perform better in terms of employability (Ricci Caballo et al., 2022). Students who opted for internships scored significantly higher than other students in adaptability and enhanced their resilience, which in turn improved their employability (Goodenough et al., 2020). Social adaptability may have little to do with specific jobs, but it is the basic work skills and thinking ability of college students, which can help them secure a job position, deal with various situations in the workplace and stand out from their work. Therefore, Hypothesis 2 was proposed:

*H2*: College students’ social adaptability has a positive effect on their employability.

### Self-efficacy

Self-efficacy refers to the judgment and perceived capability of individuals to adopt certain behaviors, complete necessary work and achieve goals under certain circumstances, which can affect the selection of tasks, the quality of task implementation, the degree of endeavor to complete selected tasks, and the perseverance in task execution (Bandura, 1997). It reflects people’s beliefs in their capabilities to learn or perform actions at a particular level, and their hope that those actions could lead to specific goals with expected results (Feltz et al., 2008). The construct has been widely applied in various research fields as it has shown high levels of correlation with learning strategies (Wang and Wu, 2008), academic performance (Choi, 2005), career success (Rigotti et al., 2020), and perseverance (Harahsheh, 2017). Self-efficacy was also identified to be positively linked to job search behavior (Moynihan et al., 2003) and acted as a significant player in graduates’ employment (Pinquart et al., 2003; Tentama and Nur, 2021). For example, the analysis of 651 college students from six provinces in China revealed that self-efficacy had a positive prediction on employability (Wang D. et al., 2022). Based on the previous studies, those with higher levels of self-efficacy would rise to the challenges in the process of acquiring knowledge and skills (Ayllón et al., 2019), have greater confidence in hunting for jobs upon graduation (Lian et al., 2021), and become more employable in the labor market (Wang D. et al., 2022). Therefore, Hypothesis 3 was put forward:

*H3*: College students’ self-efficacy positively predicts their employability.

In the face of adversity, innovators tend to demonstrate perseverance and confidence to tackle challenges and are more motivated to find ways to solve problems, which accordingly enhanced their self-efficacy (Luthans et al., 2007). Innovation passion was found to have a significant impact on employees’ abilities to free themselves from pre-defined roles to complete incorporated missions and also on employees’ self-perceived competencies fostered in long-run internal and external exchanges (Jia et al., 2021). A cross-sectional investigation of 848 nurses from eight tertiary hospitals and four secondary hospitals in Tianjin, China found that innovative behavior had a positive impact on self-efficacy (Dan et al., 2018). The SEM analysis of 339 employees and 89 supervisors of Taiwan international tourist hotels showed that creative personality had a significantly positive effect on self-efficacy in the ability to generate creative results (Teng et al., 2020).

Innovation capability was also proved to have a positive impact on self-efficacy among students. When handling new tasks, innovative students demonstrate a stronger willingness to assume risks and higher levels of self-efficacy (Pilav-Velic et al., 2020). A quantitative survey of 211 students at a large-scale public higher education institution in Southeastern America found that there was a positive association between personal innovative ability in information technology and self-efficacy in using computers in different environments (Thatcher and Perrewe, 2002). Further evidence also showed that innovation was positively related to students’ self-efficacy in programming (Liu et al., 2022) and the professional self-efficacy of undergraduate nursing students (Shen et al., 2021).

On the basis of these prior studies, the stronger innovation capability college students have, the higher self-efficacy they will be equipped with. As argued in the development of hypothesis 3, higher self-efficacy further leads to higher employability. It indicates that self-efficacy may play a mediating role in the correlation between innovation capability and employability. To test that prediction, Hypothesis 3a was formulated:

*H3a*: Self-efficacy plays a significant mediating role in the association between college students’ innovation capability and employability.

High adaptation led to a more positive self-efficacy belief and resulted in lower levels of anxiety among college students in China during the COVID-19 pandemic (Wang C. et al., 2022). A study on two samples (Sample 1 = 340, Sample 2 = 547) of college students from Thailand (Tolentino et al., 2019), a convenient sample of 358 participants from tertiary education institutions in Malaysia (Al-Jubari et al., 2021), and an online cross-sectional survey on 667 graduates in Croatia (Matijaš and Seršić, 2021) showed that adaptability had a positive relationship with self-efficacy in searching jobs. Adaptability to different contexts also had a positive prediction effect on self-efficacy in career decision-making (Ting and Datu, 2020; Kim H., 2021). Research on 14,182 science teachers and 57,131 students from 2,189 high schools across eight nations revealed that the greater teacher adaptability was, the greater teacher self-efficacy and student self-efficacy would be (Collie et al., 2020). A meta-analysis of 18 samples with a total population of 6,339 participants found that adaptability in a career context was moderately positively correlated with self-efficacy (Stead et al., 2022).

In summary, the stronger social adaptability college students have, the more self-efficacious they will be. The existing studies discussed in the development of hypothesis 3 show that higher self-efficacy resultantly relates to stronger employability. It suggests that self-efficacy may function as a mediator in the association between social adaptability and employability. Hence, Hypothesis 3b was proposed:

*H3b*: Self-efficacy plays a role in mediating the relation between social adaptability and undergraduates’ employability.

**Figure 1:**
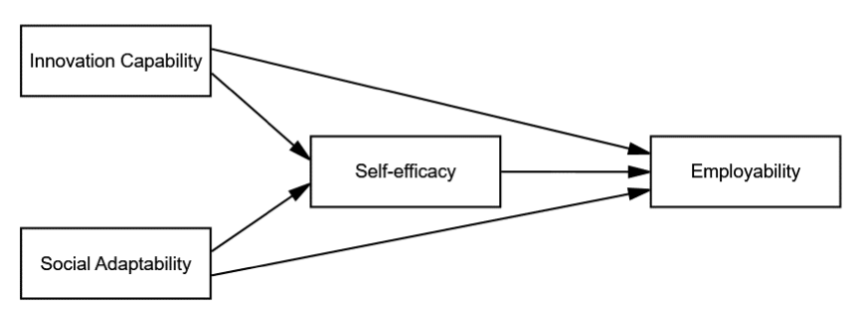
Proposed conceptual framework of the research.

The conceptual framework of the current research is shown in Figure 1. It proposes that innovation capability has a positive influence on employability. Social adaptability positively predicts employability. Self-efficacy mediates the relationship between innovation capability and employability. It also severs as a mediator in the relationship between social adaptability and employability.

## Methodology

### Pilot test

The pilot test for this study took place at Panzhihua University, a public university in Sichuan province, China. We distributed the digital version of the questionnaire to four schools, namely, School of Foreign Languages and Cultures, School of Chinese Language and Literature, School of Civil Engineering and Architecture, and School of Medicine. A total of 292 valid questionnaires were collected. We asked some respondents if there were any ambiguities or difficulties in understanding the statements or descriptions after they returned the questionnaire. To make the questionnaire more understandable, some Chinese words were revised based on suggestions from the respondents. A reliability test was performed using Cronbach’s alpha value, and exploratory and confirmatory factor analysis were conducted with SPSS 25.0 and AMOS 24. As a result, the items in the preliminary scale were reduced from 25 to 17 after the pilot test.

### Participants and sampling

A total of 729 final-year students were recruited in this study from 9 Chinese universities by convenience sampling method. A systematic review of the existing literature on developing employability skills among higher education institutions pointed out that 11 of the 13 studies adopted a convenience sampling method to collect data (Jackson and Oliver, 2018). Among the 729 participants, 124 were from Henan province (17.0%), 116 were from Guangdong province (15.9%), 91 from Shandong province (12.5%), 86 from Sichuan Province (11.8%), 79 from Hebei Province (10.8%), 64 from Hubei Province (8.8%), 60 from Anhui Province (8.2%), 50 from Jiangxi Province (6.9%), 33 from Shaanxi Province (4.5%), 20 from Hunan Province (2.7%), 4 from Tianjin municipality directly under the central government (0.5%), 1 from Fujian Province (0.1%), and 1 from Jiangsu Province (0.1%). As shown in Table 1, 422 (57.9%) were female and 307 were male (42.1%). They were all enrolled in bachelor programs in 33 disciplines such as English Language and Literature (*N* = 120), Health Service and Management (*N* = 118), Chinese Language and Literature (*N* = 114), Civil Engineering (*N* = 105), etc. The disciplines with more than 10 participants were listed in Table 1.

**Table 1:** Basic report of the sample.

Variable	Category	*N*	Percent
Gender	Male	307	42.1
	Female	422	57.9
Family Residence	Urban	391	53.6
	Rural	338	46.4
Discipline	English language and literature	120	16.5
	Health service and management	118	16.2
	Chinese language and literature	114	15.6
	Civil engineering	105	14.4
	Engineering management	60	8.2
	Translation	33	4.5
	Business English	29	4.0
	Security engineering	18	2.5
	Traffic management engineering	18	2.5
	Geomatics engineering	17	2.3
	Construction economic management	17	2.3
	Nursing	10	1.4
	Clinical medicine	10	1.4

The current study specifically focuses on the employability of final-year college students whose ages have no significant difference in China and are also of no importance to the research objectives. Thus, we did not collect information about the participants’ ages in the questionnaire survey. Previous studies on the employability of college students adopted similar data collection strategies. For example, Zhang et al. (2022) examined how university factors increase undergraduates’ employability and they did not survey the ages of the participants.

### Measures

The items used in the questionnaire in this research were all adapted from existing scales validated in the previous literature. The current questionnaire was rated on a 5-point Likert scoring system starting from 1, strongly disagree, to 5, strongly agree.

Undergraduates’ innovation capability was tested by the Scale of Influencing Factors of College Students’ Innovation Capability designed by Yang (2013) with items like “I think challenging and novel activities are very important.” The scale was tested among 1,083 students enrolled in 68 bachelor’s programs in humanities and social sciences from 11 colleges and universities and showed excellent reliability and validity. Four items were adopted in the present study after the pilot test with Cronbach’s alpha value of 0.930.

Self-efficacy was measured by The Morgan-Jinks Student Efficacy Scale (Jinks and Morgan, 1999) with items like “When the teacher asks a question, I usually know the answer even if the other students do not.” Two dimensions with 4 items were used in the current study: effort and context after the pilot test. The Cronbach’s alpha value was 0.853.

Contemporary University Students’ Social Adaptation Scale (Fang, 2008) was utilized to test social adaptability with items like “I am very concerned about the development trend of society, so as to avoid falling behind.” The scale was tested with good reliability and validity among 483 students from 7 universities in China after a small-scale pilot test. Four items were used in the current study after the pilot test. It had a Cronbach’s alpha value of 0.873.

College Students’ Employability Scale developed by He (2019) was adapted to test the construct of employability with items like “I am willing to share my information and experience with the rest of the team.” Four items were employed after the pilot test. The Cronbach’s alpha value was 0.910.

### Procedure

First, the researchers contacted teachers in the universities that participated in this study. After getting their permission, the hyperlink to the questionnaire (Praveen et al., 2019) was sent to them *via* QQ and WeChat, the most widely used network communication tools in China (Cheng et al., 2021). With their help, the questionnaire was distributed to the final-year undergraduates in the corresponding universities. Next, the students voluntarily finished the questionnaire on the online platform “Wenjuanxin” (known as China’s Qualtrics; Zhang et al., 2022) during the time span from 7 November 2021 to 25 January 2022. All participants were fully informed of the scope and objectives of the survey and the confidentiality and anonymity of their responses before filling out the measurement instrument to ensure the ethical consideration of the research. Finally, SPSS version 25.0 was used to prepare the collected data for further analysis and conduct frequency and descriptive analysis to give a basic description of the sample. SPSS Amos 24.0 was utilized to test the reliability and validity of the data as well as conduct a confirmatory factor analysis and structural equation modeling. PROCESS version 3.5 was adopted to check the mediation effect of self-efficacy.

## Results

### Reliability and validity

Cronbach’s α was applied to assess the internal consistency reliability. The rule of Cronbach’s alpha value is generally deemed: α ≥ 0.9 excellent; 0.8 ≤ α < 0.9 good; 0.7 ≤ α < 0.8 acceptable; 0.6 ≤ α < 0.7 questionable; 0.5 ≤ α < 0.6 poor; α < 0.5 unacceptable (Peterson, 1994). The entire questionnaire had excellent reliability (α = 0.939). As shown in Table 2, the construct of innovation capability (α = 0.930) and employability (α = 0.910) had excellent reliability and social adaptability (α = 0.873) and self-efficacy (α = 0.853) had good reliability. The data was suitable for factor analysis after being verified by The Kaise–Meryer–Olkin test (KMO = 0.924, Bartlett’s test of sphericity < 0.001). The items had a strong association with the corresponding construct since the factor loading of each observable variable is > 0.5 (Liao et al., 2006).

**Table 2:** Reliability and validity measures of the scale.

Constructs	Code	Mean	SD	Cronbach alpha	Factor loading	AVE	CR
IC	IC1	3.72	0.836	0.930	0.949	0.778	0.933
	IC2	3.67	0.809		0.801		
	IC3	3.66	0.842		0.793		
	IC4	3.7	0.836		0.964		
SA	SA1	3.48	0.939	0.873	0.803	0.632	0.873
	SA2	3.68	0.827		0.794		
	SA3	3.58	0.885		0.713		
	SA4	3.83	0.849		0.863		
SE	SE1	3.33	0.931	0.853	0.636	0.581	0.846
	SE2	3.12	0.9		0.659		
	SE3	3.58	0.878		0.813		
	SE4	3.38	0.881		0.813		
Emp	Emp1	3.98	0.757	0.910	0.761	0.691	0.899
	Emp2	4	0.735		0.756		
	Emp3	3.85	0.783		0.834		
	Emp4	3.89	0.798		0.847		

IC, innovation capability; SA, social adaptability; SE, self-efficacy; Emp, employability.

Construct validity was examined by convergent and discriminant validity. Average variance extracted (AVE) should be at least greater than 0.5 (Rafati et al., 2021) and the composite reliability (CR) must be greater than 0.7 (Fornell and Larcker, 1981) to indicate acceptable convergent validity. As shown in Table 2, the scale had a good convergent validity with the AVE of innovation capability, social adaptability, self-efficacy, and employability are 0.778, 0.632, 0.581, and 0.691, respectively, and the CR of each variable is 0.933, 0.873, 0.846, and 0.899. For good discriminate validity, the AVE square roots of the construct should be greater than the correlation coefficients between it and other constructs (Fornell and Larcker, 1981). Table 3 shows that the square roots of social adaptability, self-efficacy employability and employability are 0.795, 0.762, 0.831 and 0.882, respectively. The scale in this research achieved good discriminate validity since the AVE square roots of each construct exceeded the correlation coefficients with other constructs.

**Table 3:** Discriminate validity of the scale.

Items	SA	SE	Emp	IC
SA	**0.795**			
SE	0.649^***^	**0.762**		
Emp	0.733^***^	0.554^***^	**0.831**	
IC	0.717^***^	0.582^***^	0.658^***^	**0.882**

*** *p* < 0.001.

SA, social adaptability; SE, self-efficacy; Emp, employability; IC, innovation capability.

### Model estimates and hypothesis testing

Confirmatory Factor Analysis (CFA) and Structural Equation Model (SEM) were constructed by IBM SPSS AMOS 24.0. χ2/df < 5.00 was considered acceptable (Hu and Bentler, 1999; Schumacker and Lomax, 2016). RMSEA ≤ 0.05 is considered “good,” 0.05 ≤ RMSEA ≤0.08 “fair,” 0.08 ≤ RMSEA ≤ 0.10 “mediocre,” RMSEA > 0.10 “poor” (MacCallum et al., 1996). The values of GFI, CFI, NFI, TLI above 0.95 suggest excellent model fit, between 0.90 and 0.95 good (Łakuta, 2018). SRMR < 0.08 is deemed as a good fit (Hu and Bentler, 1999). Table 4 displays the results obtained from the analysis and identified that the proposed model had a good fit (χ2/df = 4.46, RMSEA = 0.069, SRMR = 0.049, GFI = 0.934, CFI = 0.965, NFI = 0.955, TLI = 0.955).

**Table 4:** Model fix index.

Fit Index	$\chi^{2}$	df	$\chi^{2}/df$	RMSEA	SRMR	GFI	AGFI	NFI	TLI	CFI
Standard	-	-	<5	<0.05	<0.05	>0.90	>0.90	>0.90	>0.90	>0.90
Model	414.773	93	4.46	0.069	0.049	0.934	0.903	0.955	0.955	0.965

**Figure 2:**
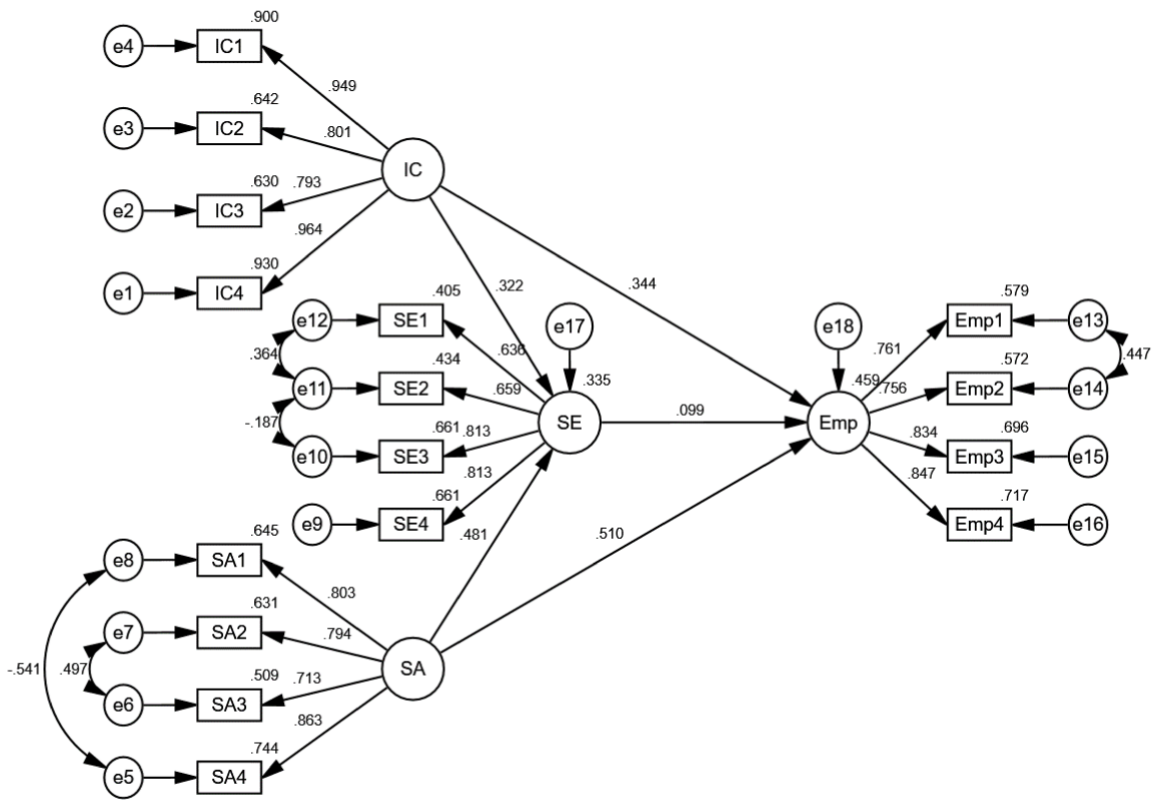
Result of the path analysis. IC, innovation capability; SA, social adaptability; SE, self-efficacy; Emp, employability.

The correlations among the latent variables were tested by SEM *via* IBM SPSS AMOS 24.0. The results are presented in Figure 2 and Table 5. The path coefficient of IC to students’ Emp was 0.344 (*p* < 0.001), thus Hypothesis 1 was supported. College students’ innovation capability positively and significantly influences their employability. When they are equipped with stronger innovation capability, they will be more employable in the job market. The path coefficient of SA to undergraduates’ Emp was 0.510 (*p* < 0.001), thus Hypothesis 2 was supported. Social adaptability of college students has a significantly positive relation to their employability. If they are more socially adaptable, they will have a better chance to find a job after graduation. The path coefficient of SE to students’ Emp was 0.099 (*p* < 0.01), thus Hypothesis 3 was supported. Self-efficacy has a positive prediction on Emp. It indicates that if college students have greater self-efficacy in their academic engagement, they will be equipped with stronger employability.

**Table 5:** Path coefficient estimates of the proposed SEM.

Hypothesis	Path	Coefficient	S.E.	C.R.	*p*-value	Test results
H1	Emp ← IC	0.344	0.025	9.288	^***^	Supported
H2	Emp ← SA	0.510	0.036	10.436	^***^	Supported
H3	Emp ← SE	0.099	0.036	2.180	0.029	Supported

*** *p* < 0.001.

IC, innovation capability; SA, social adaptability; SE, self-efficacy; Emp, employability.

The mediation effect was analyzed by using PROCESS version 3.5, an SPSS Macro (Hayes, 2018). Model 4 with Bootstrap samples of 5,000 was performed to assess indirect, direct, and total effects of the association between self-efficacy, innovation capability, social adaptability, and employability to detect the mediation effect of self-efficacy in the relations between innovation capability and employability, and between social adaptability and employability. Bootstrap CI method was set at Bias Corrected and Confidence level 95%. BootLLCI and BootULCI need to have a range that excludes 0 to establish a significant mediating effect (Flores-Barrantes et al., 2020).

Table 6 shows that the total effect of innovation capability on employability was 0.570 (*p* < 0.001) and the direct effect was 0.486 (*p* < 0.001). The indirect effect of self-efficacy on the relation between innovation capability and employability was 0.084. The range between BootLLCI (0.042) to BootULCI (0.134) excluded 0. Hence, H3a was supported. Self-efficacy plays a mediator of the effect of innovation capability on employability. Innovative college students tend to be more confident in their abilities and behaviors, which in turn increases their employability in the labor market. Self-efficacy intensifies the effect of innovation capability on employability among college students.

**Table 6:** Mediating effect of innovation capability on employability.

	Model 1 Y(Emp)			Model 2 M(SE)			Model 3 Y(Emp)		
	*Coeff.*	*SE*	p	*Coeff.*	*SE*	p	*Coeff.*	*SE*	p
X(IC)	0.570	0.026	<0.001	0.548	0.031	<0.001	0.486	0.031	<0.001
M(SE)	——	——	——	——	——	——	0.153	0.031	<0.001
Constant	1.830	0.098	<0.001	1.334	0.115	<0.001	1.626	0.105	<0.001
	$R^{2}\;$ = 0.399			$R^{2}=$ 0.306			$R^{2}=$ 0.418		
	*F*(1,727) = 481.729, *p* < 0.001			*F*(1,727) = 320.134, *p* < 0.001			*F*(2,726) = 260.839, *p* < 0.001		
Bootstrap	Indirect effect 0.084			BootLLCI 0.042			BootULCI 0.134		

IC, innovation capability; SE, self-efficacy; Emp, employability

As shown in Table 7, the total effect of social adaptability on employability was 0.579 (*p* < 0.001) and the direct effect was 0.495 (*p* < 0.001). The indirect effect of self-efficacy on the association between social adaptability and employability was 0.083. The interval of BootLLCI and BootULCI was between 0.038 and 0.133, which did not include 0. Therefore, H3b was supported. Self-efficacy played an intermediary role in the correlation between social adaptability and employability. It is more likely for the college students with higher social adaptability to have stronger beliefs in their capabilities to take action and attain expected goals, which accordingly make them more employable when seeking jobs. Self-efficacy strengthens social adaptability’s positive impact on undergraduates’ employability.

**Table 7:** Mediating effect of social adaptability on employability.

	Model 1 Y(Emp)			Model 2 M(SE)			Model 3 Y(Emp)		
	*Coeff.*	*SE*	p	*Coeff.*	*SE*	p	*Coeff.*	*SE*	p
X(SA)	0.579	0.026	<0.001	0.561	0.031	<0.001	0.495	0.031	<0.001
M(SE)	——	——	——	——	——	——	0.149	0.031	<0.001
Constant	1.824	0.098	<0.001	1.309	0.115	<0.001	1.629	0.105	<0.001
	$R^{2}$ = 0.400			$R^{2}=$ 0.312			$R^{2}=$ 0.418		
	*F*(1, 727) = 484.557, *p* < 0.001			*F*(1, 727) = 330.386, *p* < 0.001			*F*(2, 726) = 260.965, *p* < 0.001		
Bootstrap	Indirect effect 0.083			BootLLCI 0.038			BootULCI 0.133		

SA, social adaptability; SE, self-efficacy; Emp, employability.

## Discussion

The current research confirmed that innovation capability has a significant play in college students’ employability, which echoes the findings of the scarce previous studies (Okafor et al., 2020; Rees, 2021). The world is entering into an era of knowledge economy that prioritizes intellectual property, creativity, and competitive advantage. In the same manner, employers place more emphasis on building a talent pool with strong innovative spirit and ability to help them survive and prosper in the increasingly fierce market competition. As future innovators, college students are bound to intensify their innovative capabilities to get ready for the job market, maintain sustainable employment, and make contributions to economic growth and social progress. Innovation capability can turn the future workforce into problem solvers, critical thinkers, effective decision-makers, initiating managers, and constructive leaders. Previous research has revealed that innovation abilities should and can be developed in the process of engaging in higher learning (Boyles, 2012; Mayhew et al., 2016). As a result, tertiary education institutions should step up their efforts in offering integrated innovation training and education to cultivate talents that meet the needs of the stakeholders. They should strive to raise the awareness of innovation among undergraduates through related contests and entrepreneurship lectures (Zhang B. et al., 2021).

The study found a positive correlation between social adaptability and the employability of undergraduates. Social adaptability can be viewed as one of the most critical abilities for college students to step into the real world and integrate into society. They must adjust to their social surroundings, interact with other people, follow social rules, maintain social relationships, and overcome various obstacles to become social beings (Ashraf et al., 2016). At the same time, socio-cultural contexts are also significant factors that influence students’ academic achievements and work performance (Lee and Ciftci, 2014). Weak ability in socio-cultural adaptation would impede undergraduates from acquiring potential social support, lower their confidence in doing the job well and hinder their adjustment to the new environment. Although the major task of college students is to acquire the knowledge and skills required by future jobs, the ability to adjust to the changing and challenging work and social contexts and maintain good interpersonal relationships are essential guarantees for a successful career. They must learn how to reach a consensus with others while leaving aside differences and establishing favorable cooperation in the process of competition. Therefore, students need to take active participation in extra-curricular activities, join student unions or associations and take part-time jobs to develop their social skills as a necessary supplement to their employability. As for the colleges and universities, they are encouraged to integrate the cultivation of social adaptability into their teaching plans and organize more on-campus and off-campus activities to make their graduates more employable in the future labor market.

The analysis also showed that there was a significant positive association between self-efficacy and undergraduates’ employability and revealed that self-efficacy mediated the effect of innovation capability and social adaptability on employability. As previous studies have confirmed that self-efficacy has a significant and positive impact on innovative behaviors (Michael et al., 2011; Tierney and Farmer, 2011), college students should be greatly motivated to improve their self-efficacy to enhance their innovation capability and produce more innovative outcomes in the future job positions. In addition, self-efficacy was identified to be an effective mediator of the effect on students’ motivation, achievement, and performance (Zimmerman, 2000). The findings of this study were consistent with the previous research and further highlighted the critical position of self-efficacy for the development and cultivation of undergraduates as self-efficacy relates to students’ confidence in their abilities to mobilize cognitive resources for the successful completion of tasks (Bandura, 1982). If they believe that their efforts can lead to expected outcomes, they could be more engaged in academic undertakings, innovative training, and social activities. Self-efficacy encourages students to perform various actions to achieve academic and career goals and develop skills to overcome adversities in the modern job market. It would in turn help them become more confident in searching for job opportunities and pursuing excellence in workplaces. As self-efficacy has consistently been determined to have a significant impact on personal perceptions of competencies, confidence, and expectations (Michael et al., 2011), a deep comprehension of the correlation between innovation capability, social adaptability, self-efficacy, and employability can significantly improve the employment situations of college students. Therefore, tertiary education providers should intensify their efforts in reinforcing undergraduates’ self-beliefs about their abilities to innovate, adapt and achieve in interconnected and changing contexts.

**Figure 3:**
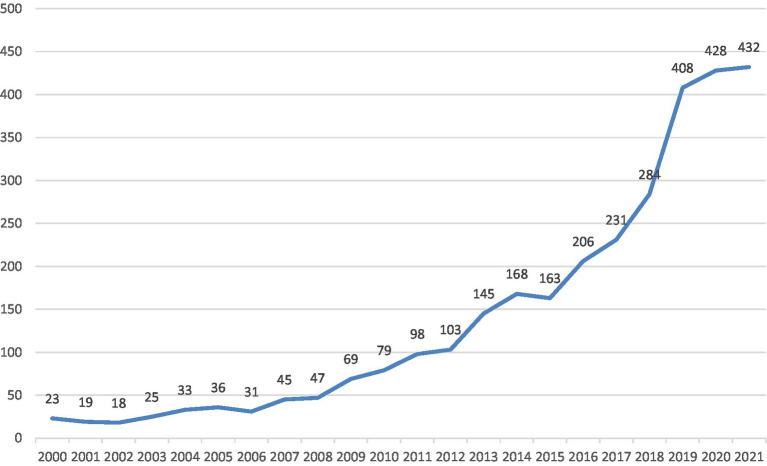
Publications of academic papers on employability between 2000 and 2021.

In terms of employability itself, the academic community is seeing the inconsistent and evolving nature of the construct’s definition. From 1 January 2000 to 31 December 2021, there were 3,091 academic references listed in the Web of Science Core Collection database (SEI-expanded, SSCI, and A&HCI) with the retrieving strategy: Topic = employability, Languages = English, Document Types = Articles or Review Articles. As shown in Figure 3, the publications on employability increased significantly in the past two decades. The total amount of published academic papers in 2021 was 18.8 times more than that in 2000, and 5.5 times more than that in 2010. Particularly, the yearly publications remained over 400 in the past 3 years, doubling the average annual amount in the period between 2016 and 2018. The consistent growing trend indicates that employability is a hot research topic in the academic community. The flourishing studies have been enriching the definition and dimension of employability. For example, the USEM model of employability developed by Knight and Yorke (2002) divided employability into four dimensions: subject understanding, skills (subject-specific and generic), personal qualities (including self-theories and efficacy beliefs), and metacognition (including reflection). Fugate et al. (2004) first proposed that employability was composed of three dimensions: career identity, personal adaptability, and social and human capital, and later examined five crucial dimensions: “openness to changes at work, work and career resilience, work and career proactivity, career motivation, and work identity” (Fugate and Kinicki, 2008). Clarke (2018) proposed an employability structure with six critical dimensions: “human capital, social capital, individual attributes, individual behaviors, perceived employability, and labor market factors.” Zhang et al. (2022) suggested that employability consists of “professional knowledge and skills, learning ability, adaptability, practical ability, communication ability, teamwork ability, information acquisition ability, and career planning ability.” Different researchers interpreted the components of employability from varying perspectives and for different research objectives, which leads to the reality that there is no consensus on the definition of employability in academia. The present study focused on the dimensions of teamwork, communication and coordination, and self-learning skill that are deemed of paramount importance to college students’ employability (He, 2019). Teamwork is the core to ensure the effective operation of the organization and all departments within the organization (Watson et al., 2022) and is a highly sought-after competence by employers (Parratt et al., 2016). A qualitative study on job developers revealed that soft skills such as communication and coordination were more desirable than job-related technical skills (Scheef et al., 2019). Self-learning skill can keep individuals updated both in knowledge and competence to maintain competitiveness in the workplace and secure sustainable employment in the contemporary lifelong learning society (Wang, 2017).

## Implications

Governments, tertiary education institutions, and employers are intensifying their contact since industries are troubled by the fact that graduates from universities and colleges are not ready for work (Edziwa and Blignaut, 2022). The results of the current study provide both theoretical and managerial implications for the possible solutions to the current employment difficulties of college graduates and suggest feasible measures for the relevant stakeholders to enhance undergraduates’ employability through a systematic reinforcement of their innovation capability, social adaptability, and self-efficacy.

### Theoretical implications

The present study examined the relations between innovation ability, social adaptability, self-efficacy, and employability and self-efficacy’s mediation in the relations between innovation capability and employability, and between social adaptability and employability of college students. By using quantitative methods including questionnaires, structural equation modeling, and mediation analysis, college students’ employability was confirmed to be significantly impacted by their innovation capability, social adaptability, and self-efficacy. Self-efficacy intensified the positive effect of innovation capability and social adaptability on undergraduates’ employability. The findings enriched the existing literature on undergraduates’ employability by revealing the psychological mechanism through which the two prominent and sought-after abilities, innovation capability and social adaptability, affect employability. The study could be of theoretical value to future researchers interested in this field to develop a more comprehensive and integrated framework to systematically investigate the factors that affect college students’ employability.

### Managerial implications

Education administration studies have become increasingly diverse and numerous over the past few years (Hallinger and Kovačević, 2019). Institutions of higher learning are faced with growing pressure to redesign their organizations to accommodate changing funding sources and social demands (Song, 2021). Universities are striving to enhance the employability of their students by fostering their innovation capability to tackle challenges at a global level (Nanjundeswaraswamy and Swamy, 2022). They are making increasing efforts to improve their teaching quality by formulating innovative practices because their students must be equipped with ample knowledge, skills, and competencies to succeed and excel in today’s ever-changing world (Asiyai, 2022). Based on the findings of the current study, higher education administrators can upgrade their employability enhancement initiatives through curriculum reform that integrates the development of innovation capability, social adaptability, and self-efficacy (Campbell et al., 2019). It accelerates the transformation of higher education management, enriches the content related to employability training, and enhances the effectiveness of human resource development. By doing so, college graduates equipped with these traits will be able to respond more quickly to the demands of industries and the challenges of the job market. They can also help organizations and governments maintain competitive advantages in the era featuring knowledge economy and innovation-driven development, which also plays an essential part in guaranteeing the sustainability of higher education.

## Conclusion

This study investigated the correlation between innovation capability, social adaptability, self-efficacy, and the employability of college students and the mediation effect of self-efficacy on the relations between innovation capability and employability and between social adaptability and employability. By adopting a quantitative approach with a questionnaire survey and conducting path analysis with structural equation modeling and mediation analysis with SPSS PROCESS Macro, we found that innovation capability, social adaptability, and self-efficacy significantly positively correlated with employability. In the meanwhile, self-efficacy functioned as a mediator in the association between innovation capability, social adaptability, and employability of undergraduates. The findings provided insight into the salient factors that impact the employability of college students, revealed the psychological mechanism through which these factors interact with employability, and proposed positive interference in students’ innovation capability, social adaptability and self-efficacy to develop their employability.

## Limitations and future research directions

The current study has made noteworthy contributions to the existing literature on undergraduates’ employability. However, it has a few limitations that can trigger future research. First, the conclusions drawn in this study revealed several valuable insights for the development of tertiary education and college students, but more research is encouraged to extend the sample size to further test the generalization of the results. Second, the study has investigated the employability of college students with the sub-factors of teamwork, communication, coordination, and self-learning skill. Future research is recommended to explore the effect of innovation capability and social adaptability on other attributes of undergraduates’ employability to grasp a more comprehensive understanding and evaluation. Third, the findings are obtained through quantitative research with a questionnaire survey as its principal research method. A qualitative approach such as interviewing the stakeholders (college students, employers) is beneficial to gain a more comprehensive understanding of the correlation between innovation capability, social adaptability, self-efficacy, and employability.

## Data availability statement

The raw data supporting the conclusions of this article will be made available by the authors, without undue reservation.

## Ethics statement

Ethical review and approval was not required for the study on human participants in accordance with the local legislation and institutional requirements. Written informed consent from the patients/participants or patients/participants legal guardian/next of kin was not required to participate in this study in accordance with the national legislation and the institutional requirements.

## Author contributions

RP: conceptualization. XL and HL: data curation. XL and RP: investigation, methodology. RP and HL: supervision and writing—review and editing. XL: writing–original draft. All authors contributed to the article and approved the submitted version.

## Funding

This research was funded by the Soft Science Project of the Science and Technology Department of Sichuan Province (grant no.: 22RKX0624).

## Conflict of interest

The authors declare that the research was conducted in the absence of any commercial or financial relationships that could be construed as a potential conflict of interest.

## Publisher’s note

All claims expressed in this article are solely those of the authors and do not necessarily represent those of their affiliated organizations, or those of the publisher, the editors and the reviewers. Any product that may be evaluated in this article, or claim that may be made by its manufacturer, is not guaranteed or endorsed by the publisher.
